# The impact of frailty syndrome on skeletal muscle histology: preventive effects of exercise

**DOI:** 10.1002/2211-5463.70049

**Published:** 2025-05-05

**Authors:** Fujue Ji, Hae Sung Lee, Haesung Lee, Jong‐Hee Kim

**Affiliations:** ^1^ Department of Physical Education, College of Performing Arts and Sport Hanyang University Seoul Korea; ^2^ BK21 FOUR Human‐Tech Convergence Program Hanyang University Seoul Korea; ^3^ Department of Physical Education, College of Education Wonkwang University Iksan Korea

**Keywords:** exercise, frailty syndrome, histology, prevent, skeletal muscle

## Abstract

Frailty syndrome, a condition marked by increased vulnerability due to age‐related physiological decline, exerts a profound impact on skeletal muscle structure and function. Despite its widespread prevalence, the underlying mechanisms contributing to frailty‐associated muscle deterioration remain poorly elucidated. This study utilized histological and biochemical analyses in a murine model to investigate the effects of frailty syndrome on skeletal muscle. Mice were classified based on age and condition, including a subset subjected to an exercise intervention. Parameters evaluated included body weight, lean mass ratio, myofiber size and number, extracellular matrix (ECM) content, and myosin heavy chain isoform expression. Frailty syndrome led to increased body weight and ECM content, coupled with reductions in myofiber size and number, reflecting substantial structural and functional impairments in skeletal muscle. Exercise interventions effectively countered these deleterious changes, preserving myofiber morphology and reducing ECM expansion, thereby demonstrating the protective role of exercise in mitigating frailty‐induced muscle deterioration. The study highlights the severe impact of frailty syndrome on skeletal muscle structure and integrity. Importantly, it underscores the potential of regular exercise as an effective therapeutic approach to prevent or reverse muscle deterioration associated with frailty, offering critical insights into managing age‐related muscular degeneration.

## Graphical abstract

Frailty syndrome exacerbates skeletal muscle degeneration via increased ECM deposition and myofiber loss. This study, using a murine model, demonstrates that endurance exercise attenuates these histopathological alterations, preserving muscle integrity. Findings support exercise as a viable strategy to counteract frailty‐induced musculoskeletal decline in aging populations.

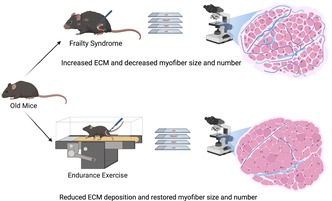

**Abbreviations**
AKT: protein kinase B
AM: adult mice
AMPK: AMP‐activated protein kinase
ARRIVE: animal research: reporting of *in vivo* experiments
CaN: calcineurin
CSA: cross‐sectional area
ECM: extracellular matrix
EOM: endurance exercise old mice
FAPs: fibro‐adipogenic progenitors
FGF: fibroblast growth factor
FS: frailty syndrome mice
H&E: hematoxylin and eosin
HYU: Hanyang University
IACUC: Institutional Animal Care and Use Committee
IL‐6: Interleukin 6
MHC: myosin heavy chain
mTOR: the mammalian target of rapamycin
NFAT: the nuclear factor of activated T cell
OM: Old mice
PGC‐1α: Pparg coactivator 1 alpha
PL: plantaris
QD: quadriceps
SDS/PAGE: sodium dodecyl sulfate–polyacrylamide gel electrophoresis
SEM: standard error of the mean
SMAD3: mothers against decapentaplegic homolog 3
TA: tibialis anterior
TEM: transmission electron microscopy
TGF‐β: transforming growth factor beta
TNF‐α: tumor necrosis factor
YM: young mice

Frailty syndrome is a clinically recognizable condition characterized by increased vulnerability resulting from age‐associated decline in physiological reserve and function across multiple organ systems. This decline compromises an individual's ability to perform daily activities and respond effectively to acute stressors, thereby increasing the risk of adverse health outcomes [1]. Frailty syndrome is typically categorized into three progressive states: pre‐frailty, frailty, and frailty‐related complications [2, 3]. Among these stages, the pre‐frailty stage is particularly critical, as individuals in this phase are highly susceptible to transitioning into frailty and developing associated complications, highlighting the progressive nature of this syndrome [2, 4].

Skeletal muscle plays a central role in whole‐body metabolism, influencing key processes such as energy balance, glucose regulation, and lipid oxidation, thereby serving as a cornerstone of metabolic health [5, 6]. Both aging and frailty impose significant detrimental effects on skeletal muscle, including reductions in muscle mass, strength, and function [3, 7, 8, 9, 10]. These changes not only exacerbate the risk of metabolic disorders such as diabetes and obesity [11, 12] but also accelerate the progression from pre‐frailty to frailty and, ultimately, frailty‐related complications, which are strongly associated with increased mortality. Despite growing evidence of frailty‐induced skeletal muscle deterioration, the precise mechanisms underlying these effects remain poorly understood.

Histological analysis offers a powerful approach to visualizing the structural and functional changes in skeletal muscle associated with frailty syndrome. For example, alterations in myofiber number and cross‐sectional area (CSA) provide insights into changes in muscle mass, power, and strength [13]. Additionally, shifts in myosin heavy chain (MHC) isoform distribution reflect metabolic adaptations in skeletal muscle, with an increased proportion of MHC I fibers indicating enhanced aerobic capacity and a higher proportion of MHC II fibers suggesting elevated glycolytic capacity [14]. Changes in the extracellular matrix (ECM) content, which provides structural support, connects myofibers, and facilitates force transmission, can also indicate significant remodeling within skeletal muscle [15]. Despite these insights, few studies have visualized the negative effects of frailty syndrome on skeletal muscle through detailed histological analysis, leaving a critical gap in understanding its pathological progression.

Endurance exercise has been shown to confer significant benefits on skeletal muscle health, including an increase in myofiber number, CSA, and strength [16, 17, 18]. Additionally, endurance exercise promotes ECM remodeling by modulating matrix metalloproteinase activity, thereby enhancing the extracellular environment and supporting overall muscle health [19, 20]. Furthermore, endurance exercise induces a metabolic shift in skeletal muscle by increasing the proportion of MHC I fibers through pathways such as AMP‐activated protein kinase (AMPK) activation and the CaN (calcineurin)/NFAT (the nuclear factor of activated T cell) signaling cascade. These pathways drive mitochondrial biogenesis and promote the transition from MHC II to MHC I isoforms in response to sustained contractile activity [21, 22]. While these findings suggest that endurance exercise exerts histological and functional benefits on skeletal muscle, the extent to which these improvements can mitigate the adverse effects of frailty syndrome remains to be fully elucidated.

This study aims to investigate the histological changes in skeletal muscle associated with frailty syndrome and to evaluate the protective effects of endurance exercise on these alterations. By providing a detailed histological characterization, this research seeks to enhance the understanding of skeletal muscle pathology in frailty syndrome and support the development of diagnostic tools for frailty‐related conditions. Moreover, the findings on the benefits of endurance exercise will contribute to the formulation of evidence‐based exercise prescriptions aimed at preventing and mitigating the progression of frailty syndrome.

## Methods

### Animals and experimental design

**Fig. 1:**
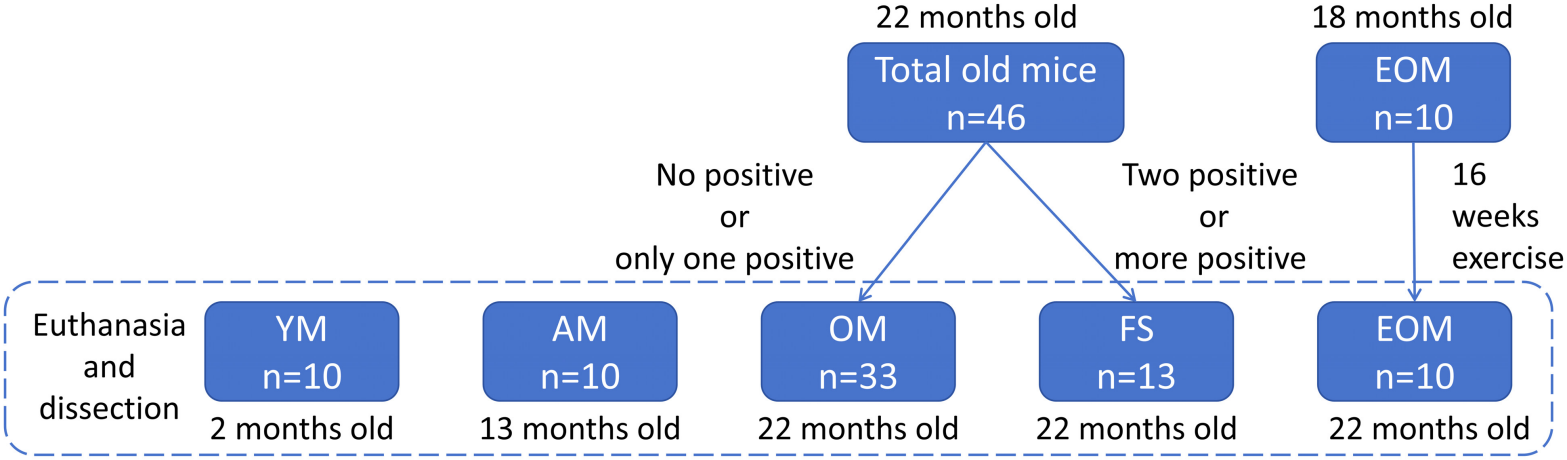
Animals and experimental design.

Male C57BL/6N mice were used as experimental subjects and divided into five groups: young (2 months old, YM, *n* = 10), adult (13 months old, AM, *n* = 10), old (22 months old, OM, *n* = 33), frailty syndrome (22 months old, FS, *n* = 13), and endurance exercise (22 months old, EOM, *n* = 10) (Fig. 1). Environmental conditions were carefully controlled with a temperature of 22 ± 2 °C, 50–60% humidity, and a 12‐h light/dark cycle. Mice had *ad libitum* access to food, water, and activity. All procedures were approved by the Institutional Animal Care and Use Committee (IACUC) of Hanyang University (HYU 2017‐0265A; 2019‐0017A) and adhered to relevant guidelines and regulations, in compliance with ARRIVE guidelines (https://arriveguidelines.org).

### Screening of frailty syndrome mouse model

**Fig. 2:**
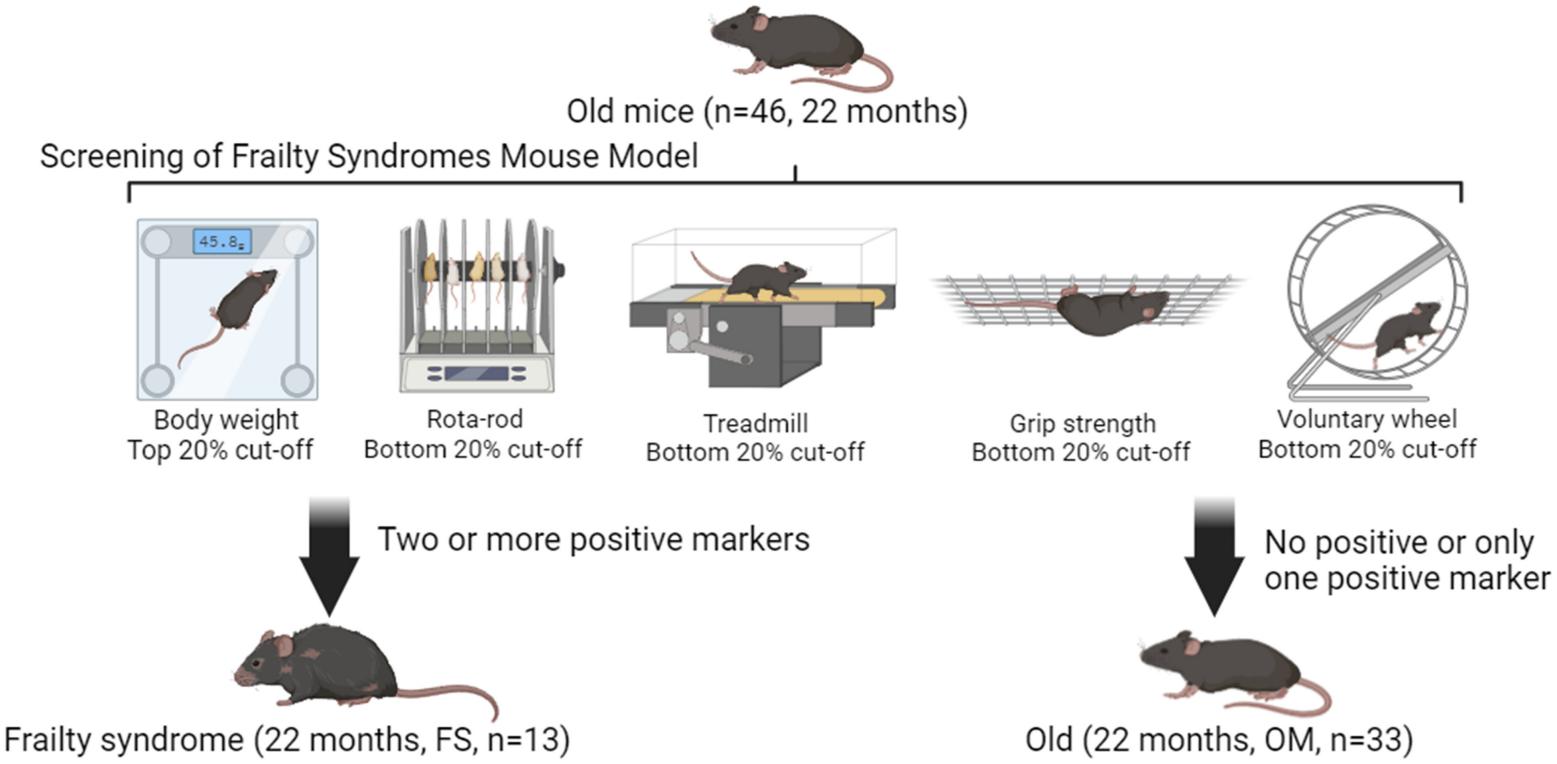
The frailty syndrome mouse model identification process.

The screening criteria for the frailty syndrome mouse model employed in this study have been extensively detailed in previous research [7, 8, 9, 10]. Briefly, 22‐month‐old mice falling within the lowest 20% for measures of slowness (Rota‐rod test), weakness (inverted‐cling grip test), poor endurance (treadmill test), or low physical activity (voluntary wheel test), or those in the highest 20% for body weight within our cohort, were identified as positive markers for frailty syndrome. These criteria were established to determine the cut‐off values for frailty syndrome (Table 1). Mice displaying two or more positive markers were classified as frailty syndrome, while those with one or no positive markers were categorized as non‐frailty syndrome [7, 8, 9, 10] (Fig. 2).

**Table 1:** Frailty syndrome criteria and cut‐off values.

Fried phenotype [23]	Mouse frailty syndrome phenotype [7, 9]	Mouse frailty syndrome cut‐off values
Low activity	Voluntary wheel test	Bottom 20% (138.60 m·day^−1^)
Poor endurance	Treadmill test	Bottom 20% (2.87 min)
Weakness	Inverted‐cling grip test	Bottom 20% (29 s)
Slowness	Rota‐rod test	Bottom 20% (55.67 s)
Lower body weight	Higher body weight	Top 20% (48.8 g)

In our previous study [3], the frailty syndrome mouse model was defined using the lowest 20% for body weight, slowness, poor endurance, weakness, and low physical activity. In this study, we revised the body weight criterion to include the highest 20% while maintaining the same thresholds for the remaining metrics. This modification aims to provide a broader interpretation of frailty syndrome, acknowledging that both increase and decrease in body weight, even within a standard diet cohort, may indicate physiological vulnerabilities. Mice with higher body weights under normal dietary conditions may exhibit traits such as reduced mobility, diminished physical endurance, and subtle metabolic inefficiencies, consistent with the multifactorial nature of frailty syndrome, which is not exclusively associated with underweight individuals [7, 8, 9, 10]. Moreover, this adjustment aligns more closely with the defining features of human frailty syndrome, which recognize both low [23] and high [24] body weight as contributing factors due to their association with impairments in mobility, strength, and systemic health. By incorporating this revised body weight criterion, our study provides a more comprehensive assessment of frailty, capturing its manifestations across a wider body weight spectrum while excluding the confounding effects of obesity or dietary interventions.

#### Body weight

Body and skeletal muscle raw weights were measured using an electronic scale (CS200; OHAUS, Parsippany, NJ, USA).

#### Slowness

Slowness was assessed using the Rota‐Rod test [3, 25]. A 3‐day adaptation phase included a pre‐test exercise at 5 rpm for 1 min, conducted once daily. The formal test employed an acceleration mode, progressively increasing speed from 5 to 50 rpm over a span of 5 min (Model 76‐0770, Harvard Apparatus Inc., Holliston, MA, USA). The latency to fall from the apparatus was recorded. Each mouse underwent three trials with a 10‐min interval between tests, and the best performance was used as the final outcome measure.

#### Weakness

Weakness was measured using the inverted‐cling grip test [3, 25]. Mice underwent an adaptation phase with a daily trial for 3 days before the official test. For the official test, each mouse was placed on the center of a wire mesh screen, and a timer was started. The screen was then inverted over the course of 2 s, positioning the mouse's head downward, and held 40–50 cm above a padded surface. The time until the mouse released its grip and fell was recorded. This measurement was repeated three times with 10‐min intervals between tests, and the longest duration was taken as the final measurement.

#### Poor endurance

Poor endurance was assessed using a treadmill test [3, 25]. Mice underwent an adaptation period for 3 days prior to the official test, running at a speed of 5 cm·s^−1^ on a 0‐degree incline for 5 min daily. For the official test, the treadmill speed began at 5 cm·s^−1^ and increased by 1 cm·s^−1^ every 20 s, maintaining a 0‐degree incline. The test ended when the mouse contacted the shock pad (set at 0.5 mA) three times.

#### Low activity

Low activity was measured using the voluntary wheel test [3, 25]. Running distance was monitored using a voluntary wheel apparatus (MAN86130, Lafayette Instrument Company, Lafayette, IN, USA), where each wheel rotation corresponded to 0.4 m. The average running distance over a 5‐day period was used as the final measurement.

### Exercise protocol

**Fig. 3:**
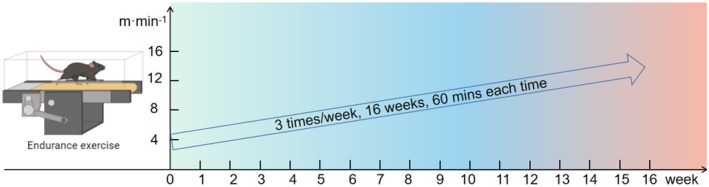
Endurance exercise protocol for endurance exercise old mice (EOM).

The EOM group (18 months old) underwent a 16‐week endurance exercise regimen. At the end of the endurance exercise period, the mice (22 months old) were euthanized. The EOM group was subjected to endurance exercise on a treadmill, with a preparatory adaptation phase conducted 1 week prior to the main experiment, during which all aged mice underwent three sessions of acclimatization at an intensity of 5 m·min^−1^ for 20 min per session. Following the start of the formal experiment, mice exercised for 60 min, three times a week, for 16 weeks. Exercise intensity was gradually increased from 4 to 16 m·min^−1^ per week and subsequently maintained, with the treadmill incline set at 0°. Instead of using electrical stimulation, the mice were gently guided to run naturally by using a wooden stick as needed (Fig. 3).

### Preservation and preparation of skeletal muscle

All mice were euthanized by cervical dislocation under deep anesthesia induced with ketamine (40 mg·kg^−1^) and medetomidine (0.8 mg·kg^−1^) to ensure loss of consciousness prior to euthanasia [26]. After the mice were euthanized, the tibialis anterior (TA), quadriceps (QD), and plantaris (PL) were carefully removed. The left leg muscles were fixed in 10% formalin for 24 h, then dehydrated and embedded in paraffin. The sections were prepared for future hematoxylin and eosin (H&E) and Masson's trichrome staining. The right leg muscles were weighed, frozen in liquid nitrogen, and subsequently stored at −80 °C for subsequent MHC isoform analyses.

### Masson's trichrome staining and analysis

Paraffin‐embedded tissues were sectioned at 5 μm. After deparaffinization and rehydration, the tissue sections were stained using Masson's trichrome staining kit (G1340, Beijing Solarbio Science & Technology Co., Ltd., Beijing, China). The sections were subsequently placed in xylene and alcohol for dehydration and transparency and finally sealed with neutral resin. Images were acquired using a slide scanner (Axio Scan. Z1, Zeiss, Jena, Thüringen, Germany) at 200× magnification. For each mouse, a single section of each skeletal muscle was randomly photographed to obtain 5 images, each covering an area of 0.271 square millimeters. The average of these five images was used to represent the ECM value of that skeletal muscle for the mouse. imagej software was used for analysis. A threshold was established to remove the blank background; the ECM signal was isolated using color deconvolution and thresholding, and the area of the ECM signal was subsequently compared with the net area of the muscle section using imagej software (National Institutes of Health, Bethesda, MD, USA).

### H&E staining and analysis

H&E staining was performed on paraffin‐embedded tissue sections after deparaffinization and rehydration, as previously described by Robert D. Cardiff [27], with slight modifications. Briefly, rehydrated tissue sections were stained using ClearView™ hematoxylin (MA0101010, StatLab) for 3 min, rinsed in tap water for 30 s, differentiated in 1% hydrochloric acid alcohol for 5 s, washed in tap water for 3 min, and stained in ClearView™ eosin (MA0101015, StatLab, Mckinney, TX, USA) for 1 min. They were subsequently dehydrated, rendered transparent, and sealed with neutral resin. Images were acquired using a slide scanner (Axio Scan. Z1, Zeiss) at 200× magnification. For each mouse, a single section of each skeletal muscle was randomly photographed to obtain 5 images, each covering an area of 0.271 square millimeters. The average of these five images was used to represent the CSA and fiber number of that skeletal muscle for the mouse. The CSA and fiber number were quantitatively analyzed using Cellpose [28, 29].

### Electrophoretic separation of MHC isoforms

The MHC isoform composition of each fiber was determined using SDS/PAGE and silver staining. Skeletal muscle was extracted for 60 min on ice in 50 vol buffer (0.1 m sodium phosphate, pH 7.3), to which a 1 : 100 proteases + phosphatase inhibitor cocktail (WSE‐7420, ATTO) was added. The total protein concentration was subsequently determined using the Pierce™ BCA Protein Assay Kit (23227, Thermo, Boston, MA, USA). Samples were diluted in 5× sample buffer [25% β‐MEtOH (β‐ME), 11.5% SDS, 50% glycerol, 312.5 mm Tris, pH 6.8, and 0.05% bromophenol] and dH_2_O to achieve a final protein concentration of 0.266 μg·μL^−1^. The samples were subsequently boiled for 10 min, and 6 μg (30 μL) of protein was loaded onto a mini gel system (Mini Protean III: 8.3 × 7.3 cm). MHC isoforms were separated using 8% SDS/PAGE with 30% glycerol [30]. The lower running buffer comprised 0.05 m Tris (base), 75 mm glycine, and 0.05% w/v SDS. The upper running buffer was 6× the concentration of the lower running buffer, and β‐ME was added (final concentration: 0.2% v/v) [31]. After sample loading, electrophoresis was performed at a constant voltage of 140 V for 28 h. The temperature of the buffer was maintained at 4 °C during electrophoresis. After electrophoresis, the gels were stained with a silver staining kit (24612, Thermo), and the bands were quantified via densitometry using imagej software.

### Statistical analysis

Analyses were performed using graphpad prism (version 9, GraphPad Software LLC, Boston, MA, USA) software. One‐way analysis of variance and Tukey's *post‐hoc* test was used to compare the mean difference among groups. When the conditions of normality or homogeneity were not met in comparisons of three or more groups, the Kruskal–Wallis test along with Dunn's *post‐hoc* comparison was employed. All results are expressed as the mean ± standard error of the mean (SEM). Statistical significance was set at *P* < 0.05. Asterisks indicate the following: *P < 0.05, **P < 0.01, ***P < 0.001, and ****P < 0.0001.

## Results

### Endurance exercise prevents frailty syndrome by reducing body weight

**Fig. 4:**
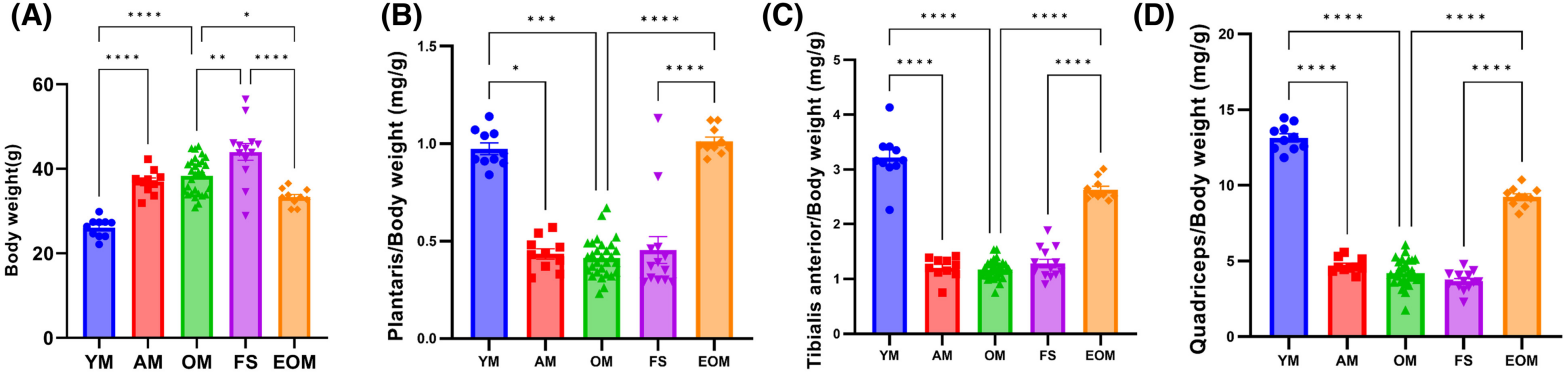
Body weight and lean muscle ratio. (A) Body weights of the different groups of mice. (B–D) Plantaris (PL), tibialis anterior (TA), and quadriceps (QD) to body weight ratios. Young mice (YM) = 2 months old, *n* = 10; Adult mice (AM) = 13 months old, *n* = 10; Old mice (OM) = 22 months old, *n* = 33; Frailty syndrome mice (FS) = 22 months old, *n* = 13; and endurance exercise old mice (EOM) = 22 months old, *n* = 10. The Kruskal–Wallis test along with Dunn's *post‐hoc* comparison was employed. All data are presented as the mean ± standard error of the mean (SEM) (*P < 0.05, **P < 0.01, ***P < 0.001, ****P < 0.0001).

Aging leads to an increase in body weight, and the frailty syndrome further exacerbates this phenomenon (*P* < 0.05) (Fig. 4A). Aging also reduces the lean mass ratio (*P* < 0.05), but the frailty syndrome does not (*P* > 0.05) (Fig. 4B–D). After 16 weeks of endurance exercise, the EOM group showed significant decreases in body weight and increases in the lean mass ratio compared to the OM and FS groups (*P* < 0.05) (Fig. 4A–D). These data suggest that aging leads to weight gain, and the frailty syndrome exacerbates this phenomenon but does not significantly alter the lean mass ratio. Importantly, 16 weeks of endurance exercise can prevent frailty syndrome by reducing body weight.

### Endurance exercise prevents frailty syndrome by reducing ECM content

**Fig. 5:**
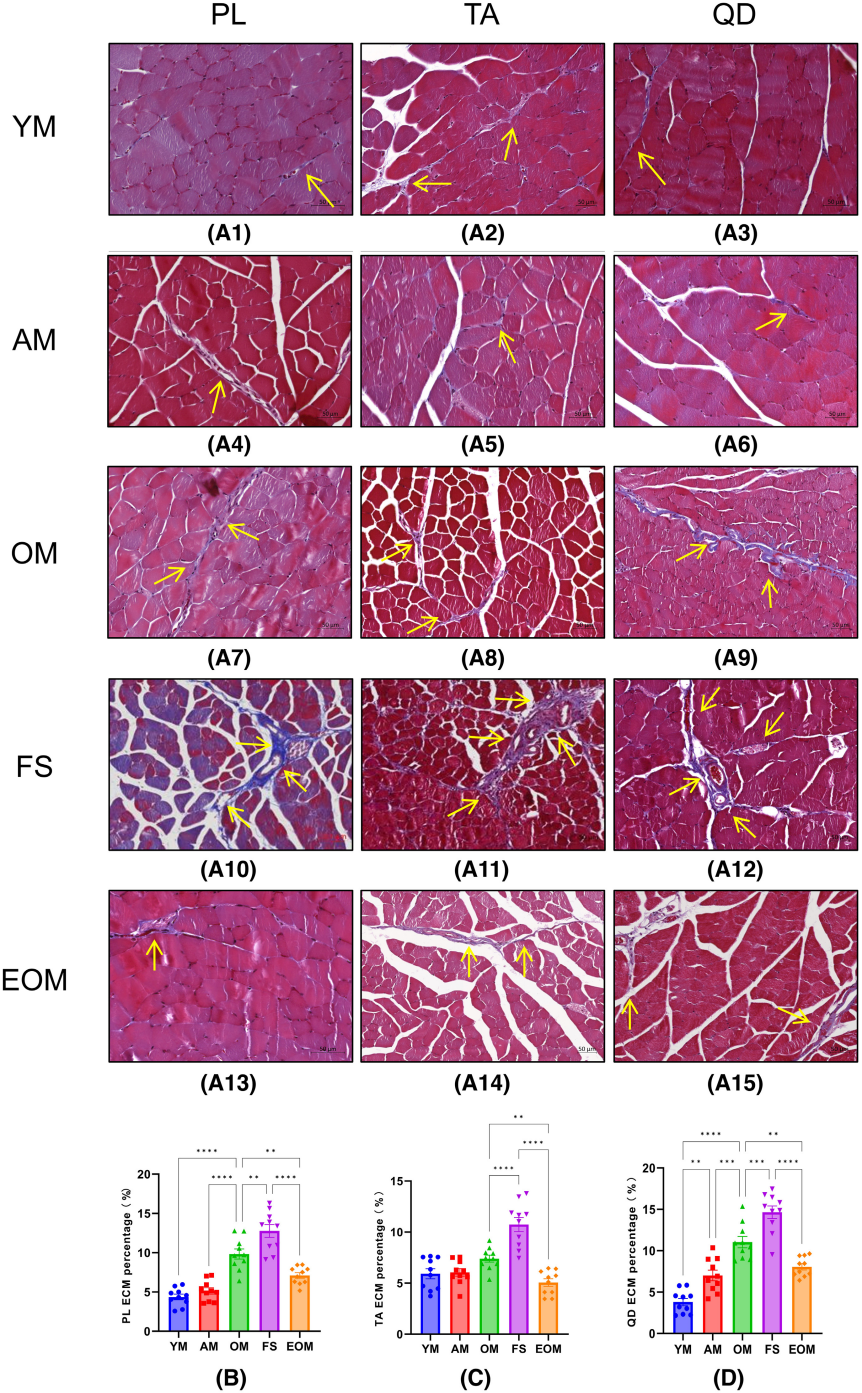
Representative images of Masson's trichrome staining of the plantaris, tibialis anterior, and quadriceps extracellular matrixes of male C57BL/6N mice at different stages. (A) Representative images of Masson's trichrome staining. (B) Changes in the extracellular matrix of the plantaris in male C57BL/6N mice. (C) Changes in the extracellular matrix (ECM) of the tibialis anterior in male C57BL/6N mice. (D) Changes in the ECM of the quadriceps in male C57BL/6N mice. Young mice (YM) = 2 months old, *n* = 10; Adult mice (AM) = 13 months old, *n* = 10; Old mice (OM) = 22 months old, *n* = 10; Frailty syndrome mice (FS) = 22 months old, *n* = 10; and endurance exercise old mice (EOM) = 22 months old, *n* = 10. ECM content is indicated in blue and the myofibers in red, labeled the ECM with a yellow arrow. The Kruskal–Wallis test along with Dunn's *post‐hoc* comparison was employed. All data are presented as the mean ± standard error of the mean (SEM) (**P < 0.01, ***P < 0.001, ****P < 0.0001). Scale bar = 50 μm.

Experimental results show that the ECM content of the PL (Fig. 5A1,A4,A7) and QD (Fig. 5A3,A6,A9) increases with age (*P* < 0.05) (Fig. 5B,D), while the TA (Fig. 5A2,A5,A8) does not appear to be affected by age (*P* > 0.05) (Fig. 5C). The impact of frailty syndrome on skeletal muscle ECM content is significant. In the PL (Fig. 5A7,A10), TA (Fig. 5A8,A11), and QD (Fig. 5A9,A12), the ECM content in the FS group is significantly larger than in the OM group (*P* < 0.05) (Fig. 5B–D). Additionally, after 16 weeks of endurance exercise, the ECM in the EOM group is significantly lower than in the OM and FS groups (*P* < 0.05) (Fig. 5A7–A15,B–D). These findings indicate that aging is a key factor leading to abnormal ECM content accumulation in skeletal muscle, and this negative effect is significantly exacerbated by frailty syndrome. Importantly, 16 weeks of endurance exercise can prevent frailty syndrome by inhibiting abnormal ECM content accumulation.

### Endurance exercise prevents decreased CSA due to frailty syndrome

**Fig. 6:**
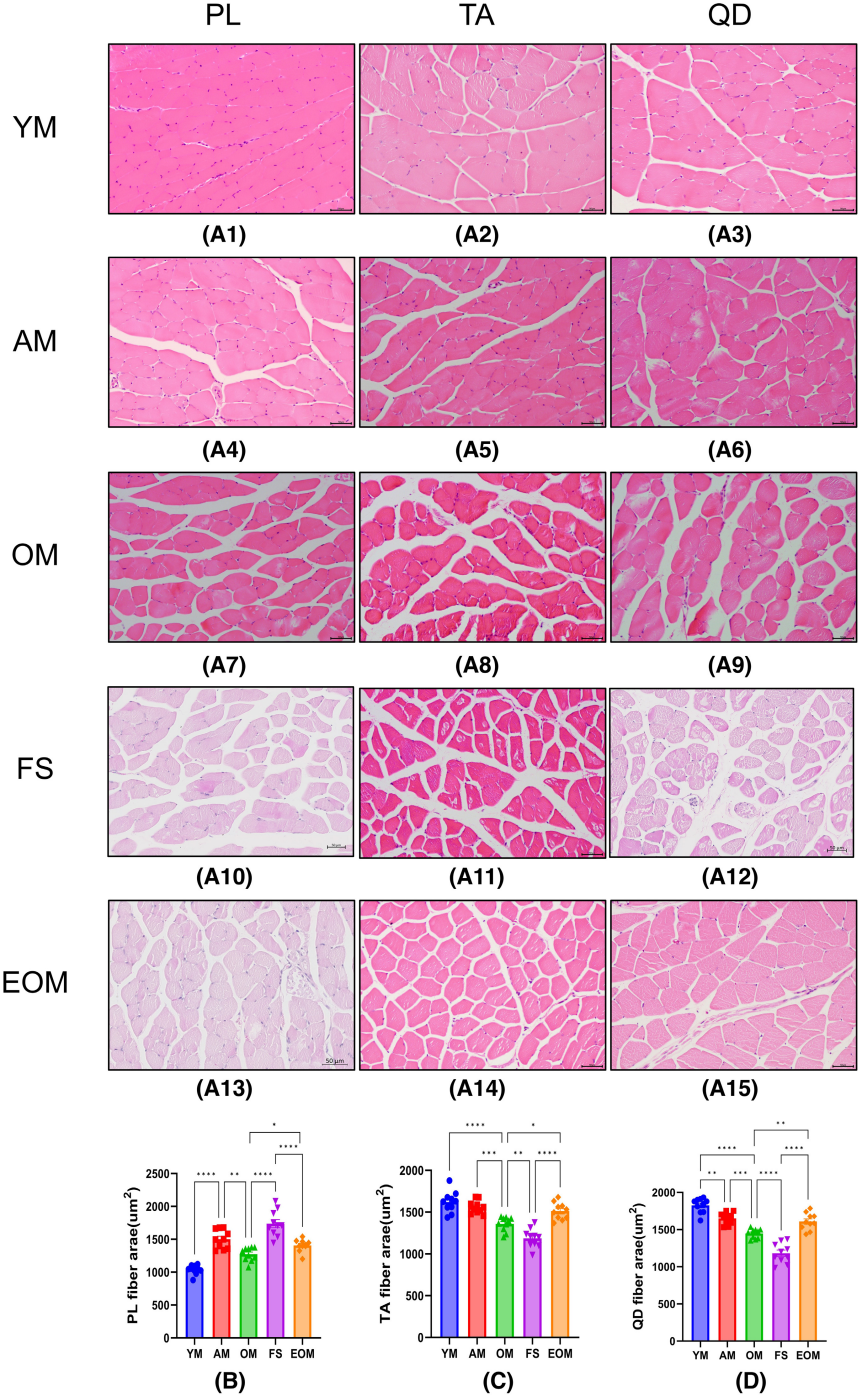
Representative images showing Hematoxylin and eosin (H&E) staining of Plantaris (PL), tibialis anterior (TA), and quadriceps (QD) cross‐sectional areas (CSA) in male C57BL/6N mice at various stages. (A) Representative images of H&E staining. (B) Changes in the cross‐sectional area of the plantaris in male C57BL/6N mice. (C) Changes in the cross‐sectional area of the tibialis anterior in male C57BL/6N mice. (D) Changes in the cross‐sectional area of the quadriceps in male C57BL/6N mice. Young mice (YM) = 2 months old, *n* = 10; Adult mice (AM) = 13 months old, *n* = 10; Old mice (OM) = 22 months old, *n* = 10; Frailty syndrome mice (FS) = 22 months old, *n* = 10; and endurance exercise old mice (EOM) = 22 months old, *n* = 10. The Kruskal–Wallis test along with Dunn's *post‐hoc* comparison was employed. All data are presented as the mean ± standard error of the mean (SEM) (*P < 0.05, **P < 0.01, ***P < 0.001, ****P < 0.0001). Scale bar = 50 μm.

The effects of aging on the CSA of different skeletal muscles vary, which may be determined by the functional roles in daily activities. We observed that in the TA (Fig. 6A8,A11) and QD (Fig. 6A9,A12), the CSA of the FS group was significantly lower than that of the OM group (*P* < 0.05) (Fig. 6C,D). In contrast, in the PL (Fig. 6A7,A10), the CSA of the FS group was significantly higher than that of the OM group (*P* < 0.05) (Fig. 6B). Additionally, after 16 weeks of endurance exercise, the CSA of the EOM group was significantly higher than that of the OM and FS groups in TA (Fig. 6A8,A11,A14) and QD (Fig. 6A9,A12,A15) (*P* < 0.05), but not PL (Fig. 6A7,A10,A13) (*P* > 0.05) (Fig. 6B–D). These data demonstrate that the impact of frailty syndrome on skeletal muscle CSA varies; 16 weeks of endurance exercise can partially prevent the decline in skeletal muscle CSA caused by frailty syndrome.

### Endurance exercise prevents decreased myofiber number due to frailty syndrome

Data show that aging leads to a significant reduction in the myofiber number, and frailty syndrome further exacerbates this decrease (*P* < 0.05) (Figs 6A1–A12 and 7A–C). Additionally, 16 weeks of endurance exercise EOM increased the myofiber number, significantly surpassing both the FS and OM groups (*P* < 0.05) (Figs 6A7–A15 and 7A–C). These experimental results reveal that frailty syndrome reduced the myofiber number; 16 weeks of endurance exercise can prevent the negative effects of frailty syndrome on skeletal muscle by increasing the number of myofibers.

**Fig. 7:**
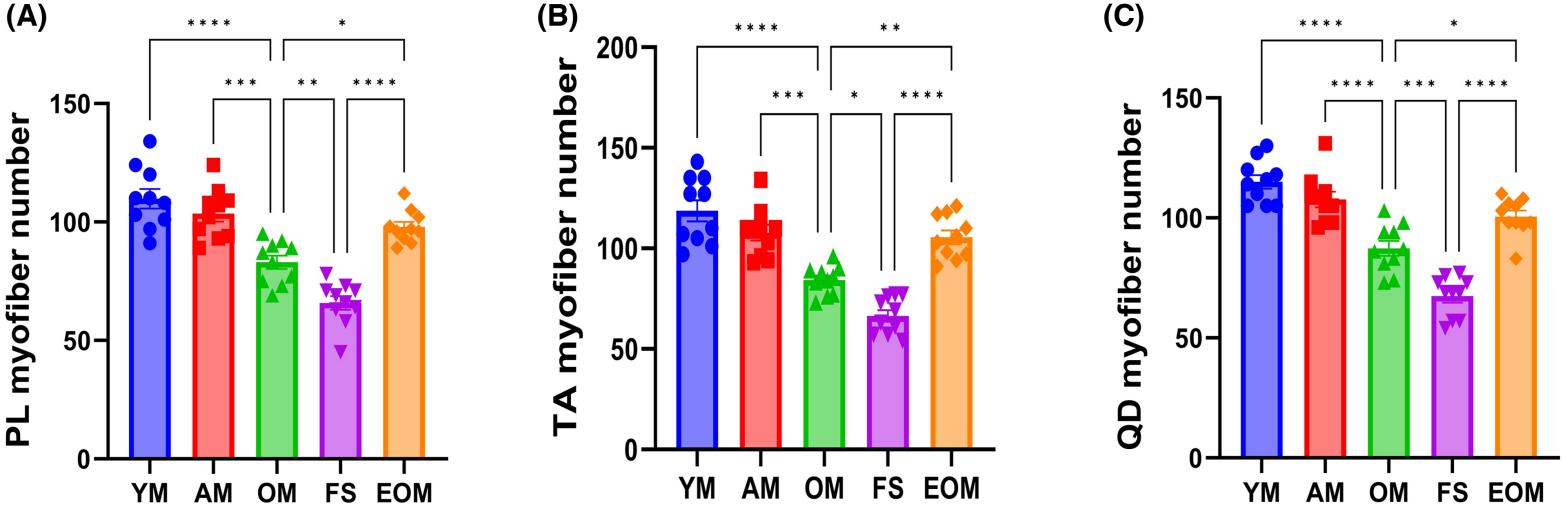
Changes in the myofiber number of the plantaris (PL), tibialis anterior (TA), and quadriceps (QD) in male C57BL/6N mice at different stages. (A) Changes in the myofiber number of the plantaris in male C57BL/6N mice. (B) Changes in the myofiber number of the tibialis anterior in male C57BL/6N mice. (C) Changes in the myofiber number of the quadriceps in male C57BL/6N mice. Young mice (YM) = 2 months old, *n* = 10; Adult mice (AM) = 13 months old, *n* = 10; Old mice (OM) = 22 months old, *n* = 10; Frailty syndrome mice (FS) = 22 months old, *n* = 10; and endurance exercise old mice (EOM) = 22 months old, *n* = 10. The Kruskal–Wallis test along with Dunn's *post‐hoc* comparison was employed. All data are presented as the mean ± standard error of the mean (SEM) (*P < 0.05, **P < 0.01, ***P < 0.001, ****P < 0.0001).

### Frailty syndrome does not affect MHC isoforms

**Fig. 8:**
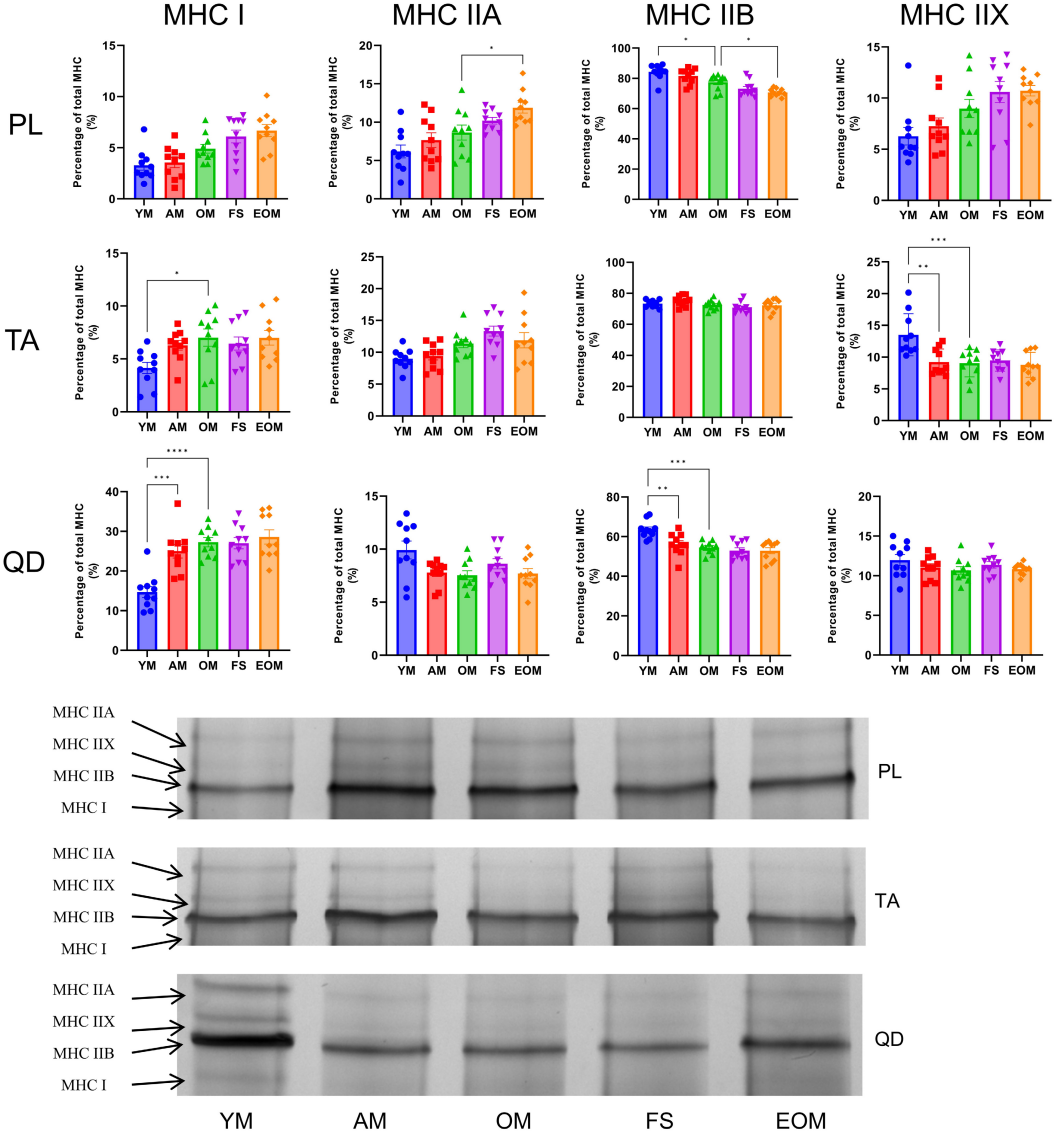
Changes in the myosin heavy chain isoform percentage of the plantaris (PL), tibialis anterior (TA), and quadriceps (QD) to body weight ratios in male C57BL/6N mice at different stages. Young mice (YM) = 2 months old, *n* = 10; Adult mice (AM) = 13 months old, *n* = 10; Old mice (OM) = 22 months old, *n* = 10; Frailty syndrome mice (FS) = 22 months old, *n* = 10; and endurance exercise old mice (EOM) = 22 months old, *n* = 10. The Kruskal–Wallis test along with Dunn's *post‐hoc* comparison was employed. All data are presented as the mean ± standard error of the mean (SEM) (*P < 0.05, ** *P* < 0.01, ***P < 0.001, ****P < 0.0001).

We used an anti‐MHC (Myosin 4 Monoclonal Antibody, Thermo, #14‐6503‐82) to examine the band positions in the gel, confirming that the bands observed in silver staining correspond to MHC (Figs S1 and S2). Aging leads to an increase in the proportion of MHC I and a decrease in the proportion of MHC IIX in TA (*P* < 0.05) (Fig. 8). Similarly, in QD, aging significantly increases the proportion of MHC I (*P* < 0.05) (Fig. 8). We also found that frailty syndrome does not significantly affect the proportion of MHC in skeletal muscle (*P* > 0.05) (Fig. 8). Sixteen weeks of endurance exercise resulted in a significant decrease in the proportion of MHC IIB only in TA (*P* < 0.05) (Fig. 8). These data indicate that frailty syndrome does not affect the proportion of MHC in skeletal muscle.

## Discussion

The detrimental effects of frailty syndrome on skeletal muscle health have been well documented [3, 7, 9, 10], yet the underlying mechanisms and effective preventive strategies remain insufficiently understood. Addressing this knowledge gap is critical, as understanding these mechanisms and identifying effective preventive measures could offer valuable insights for managing and preventing aging‐related diseases. Our study demonstrated that frailty syndrome is associated with increased body weight and ECM deposition, along with reductions in CSA and myofiber number in skeletal muscle. Notably, endurance exercise was shown to mitigate these adverse effects, underscoring its potential as a protective intervention. These findings provide a robust foundation for future research focused on the prevention, diagnosis, treatment, and post‐recovery management of frailty syndrome.

### Appropriateness of a frailty syndrome mouse model

In this study, we adopted the frailty syndrome mouse model selection criteria outlined by Thompson et al. and validated in several studies [7, 9, 10]. This approach is widely used in preclinical research on frailty syndrome [3, 7, 8, 9, 10, 32]. Unlike the Fried phenotype framework for frailty syndrome in humans [33], which includes unintentional weight loss as a diagnostic criterion, our model identifies older mice with high body weight, specifically those in the top 20% weight percentile, as positive indicators of frailty syndrome. This classification is supported by several compelling arguments. First, elevated body weight has been shown to negatively impact lifespan in mammals [34]. For instance, Ames dwarf mice, characterized by reduced body weight, exhibit increased longevity [35, 36], while calorie restriction, which reduces body weight, is associated with extended average and maximum lifespan [37, 38]. Furthermore, a positive correlation between higher body weight and increased mortality rates has been demonstrated in mice [39, 40]. These findings highlight that high body weight poses a greater threat to health in mice than low body weight, which aligns more closely with the negative health consequences typically associated with frailty syndrome [8]. Additionally, the 20% weight cut‐off point used in our study corresponds to the percentile established for the diagnosis of frailty syndrome in humans [33] and has been similarly applied in rodent models of frailty syndrome [3, 7, 9, 10]. Details of our selection criteria and methodology are provided in the Table S1.

Using 22‐month‐old mice in this study allowed for a closer approximation of human frailty syndrome characteristics, as this age corresponds to approximately 60–65 years in humans [41, 42]. This age range represents a critical period when frailty syndrome is commonly observed during the natural aging process [33, 43]. Moreover, frailty syndrome is thought to be reversible, and this age range provides a sufficient window for potential interventions, such as endurance exercise, to mitigate or reverse the progression of frailty.

Previous studies have classified individuals with two positive frailty criteria as being in the pre‐frail stage. However, in this study, frailty syndrome was defined as the presence of two or more positive criteria, a classification that more accurately reflects the continuum of frailty progression. Frailty is a progressive condition typically categorized into three stages: pre‐frailty, frailty, and frailty‐related complications [2, 44]. Given that frailty is associated with significant health risk, early detection is crucial for timely intervention. Research indicates that individuals in the pre‐frailty stage are at a higher risk of progressing to frailty and developing frailty‐related complications compared to those without pre‐frailty characteristics [4]. However, the pre‐frailty stage is also a critical window for potential reversal, as appropriate interventions can help restore functional capacity and delay further deterioration [4]. In contrast, at the advanced stage of frailty‐related complications, the condition substantially contributes to morbidity and mortality, underscoring the need for early intervention strategies [45]. By defining frailty syndrome as meeting two or more positive markers, this study aimed to capture even mild functional impairments, aligning with the human pre‐frailty classification proposed by Fried et al. [33]. This approach enhances the clinical relevance of the findings, facilitating better identification of individuals at risk. Furthermore, given that certain markers, such as increased body weight and reduced grip strength, are strongly correlated with frailty progression, adopting this threshold ensures that key frailty characteristics are not overlooked.

### Lean mass ratio in frailty syndrome

Although aging is commonly associated with a decline in lean mass ratio, our study revealed no significant differences in this parameter between the old mice (OM) and frailty syndrome (FS) groups. This unexpected result may be attributed to the high body weight characteristic of the frailty syndrome mouse model, which likely promotes ectopic fat deposition within skeletal muscle, a condition known as myosteatosis. Myosteatosis, or fat infiltration of skeletal muscle, is a recognized hallmark of metabolic and functional impairment in aging skeletal muscle. This observation aligns with clinical studies that have reported the presence of myosteatosis in the skeletal muscle of patients with frailty syndrome [46, 47]. Moreover, increased ECM content within skeletal muscle, which is often observed in frailty syndrome, may exacerbate fat infiltration, further contributing to the apparent preservation of skeletal muscle mass.

We hypothesize that this fat infiltration associated with high body weight could result in an overestimation of skeletal muscle mass, potentially explaining the absence of a decline in lean mass ratio in frailty syndrome. Additionally, frailty syndrome mice may experience prolonged weight‐bearing stress on skeletal muscles imposed by higher body weight, necessitating compensatory adaptations. These adaptations may include increases in skeletal muscle strength and structural modifications to support essential activities such as postural maintenance. Similar compensatory mechanisms have been previously reported in the context of high body weight [48, 49].

Importantly, our study demonstrates the preventive benefits of endurance exercise against frailty syndrome. Endurance exercise not only reduced body weight but also significantly improved the lean mass ratio, indicating its potential to counteract adverse changes associated with frailty syndrome. This finding underscores the efficacy of endurance exercise as a non‐pharmacological intervention to mitigate the effects of frailty syndrome and maintain skeletal muscle health in aging populations.

### ECM in frailty syndrome

Our results revealed a significant increase in ECM content in the FS group compared to the OM group, representing the first report of this phenomenon. Although the direct impact of frailty syndrome on ECM dynamics remains incompletely understood, previous studies have demonstrated that both aging [50, 51] and high body weight [52] are associated with elevated ECM deposition. The abnormal increase in the ECM observed in frailty syndrome may result from the combined effects of these factors, promoting the excessive deposition of ECM components such as collagen and fibrinogen through mechanisms involving oxidative stress and chronic inflammation [53]. These processes are likely mediated by dysregulated matrix metalloproteinase activity and the activation of hypoxia‐inducible factors, further contributing to pathological ECM remodeling [54]. Moreover, Fibro‐adipogenic progenitors (FAPs), a population of mesenchymal progenitor cells essential for muscle regeneration, become dysregulated in frailty, driving excessive ECM deposition and fibrosis via the TGF‐β/SMAD3 signaling pathway. This process contributes to increased muscle stiffness and impaired function [55, 56]. Under pathological conditions such as frailty syndrome, FAPs may aberrantly differentiate into adipocytes, exacerbating myosteatosis. This process is further amplified by the presence of inflammatory cytokines, including IL‐6 and TNF‐α [57, 58].

Excessive ECM accumulation is clinically significant, as it may exacerbate fat infiltration in skeletal muscle, thereby influencing the lean mass ratio and impairing muscle function in frailty syndrome. Our findings are indirectly supported by clinical research demonstrating that frail patients exhibit elevated levels of fibroblast growth factor (FGF) and follistatin, both of which are crucial regulators of muscle metabolism and fibrosis [59]. Furthermore, emerging evidence indicates that certain cytokines may play a pivotal role in the progression of muscle fibrosis and could serve as potential biomarkers for monitoring frailty status [60]. Consequently, our findings propose that an abnormal increase in ECM content may serve as a novel histological marker for skeletal muscle affected by frailty syndrome, providing potential diagnostic and therapeutic insights.

Importantly, our study also highlights the beneficial role of endurance exercise in mitigating frailty syndrome by reducing skeletal muscle ECM content in aging individuals. Evidence from previous studies supports that endurance exercise can decrease ECM deposition and promote ECM remodeling, enhancing skeletal muscle health [61, 62]. A well‐regulated ECM is essential for efficient muscle force transmission [62], mechanical stability of nerves and blood vessels [63], and facilitation of satellite cell proliferation and differentiation [62]. An impaired ECM state may be a contributing factor to frailty syndrome. These findings suggest that maintaining ECM homeostasis through regular endurance exercise may play a pivotal role in mitigating the progression of frailty syndrome.

### CSA in frailty syndrome

Unexpectedly, frailty syndrome mice exhibited an abnormal increase in the CSA of the PL muscle, a finding that has not been previously reported in the literature on frailty syndrome. We hypothesize that this increase may result from a reduced proportion of smaller myofibers within the PL muscle, thereby elevating the mean fiber area. Alternatively, this phenomenon could represent a compensatory adaptation aimed at mitigating the decline in muscle strength and preserving essential postural and functional activities, as suggested by prior research [63, 64]. Furthermore, frailty syndrome mice displayed increased resting time and reduced physical activity, thereby contributing to a sedentary lifestyle (S1). Given that the PL muscle is more heavily engaged in postural support than the QD and TA muscles [65, 66], this compensatory response may specifically explain the observed CSA increase in the PL, a phenomenon not apparent in the QD and TA muscles.

In contrast, frailty syndrome induced a significant reduction in the CSA of QD and TA muscles, which is likely attributable to various pathological mechanisms. Frailty syndrome is known to reduce myofiber CSA through several biological processes. First, dysregulated autophagy in frailty syndrome impairs the clearance of damaged skeletal muscle proteins, leading to a reduction in myofiber CSA [67, 68]. Second, an imbalance between protein synthesis and degradation, commonly observed in aging‐associated frailty syndrome, further exacerbates the reduction in myofiber CSA [69]. Third, increased oxidative stress and chronic inflammation in individuals with frailty syndrome contribute to skeletal muscle cell damage, further contributing to decreased CSA [70]. Collectively, these findings suggest that a significant reduction in CSA is a hallmark histological characteristic of frailty syndrome in skeletal muscle. In addition, skeletal muscle myosteatosis may further exacerbate CSA reduction by promoting pathological tissue remodeling. Adipose tissue functions as an active endocrine organ, secreting pro‐inflammatory cytokines (TNF‐α, IL‐6) and adipokines (leptin, resistin, visfatin), which contribute to muscle fibrosis, fiber atrophy, and mitochondrial dysfunction [54]. This inflammatory process, termed inflammaging, is particularly relevant in frailty syndrome, as chronic low‐grade inflammation accelerates muscle degradation and impairs regenerative capacity [71]. The observed increase in ECM deposition in frailty syndrome mice supports this notion, suggesting that persistent inflammation and pathological ECM remodeling play a crucial role in frailty‐associated muscle deterioration.

Our findings further demonstrated that endurance exercise significantly increased myofiber CSA, consistent with findings from previous research [72, 73, 74, 75]. Although endurance exercise is not traditionally associated with muscle hypertrophy, long‐term endurance training performed at appropriate intensities has been shown to increase myofiber CSA [76]. Mechanistically, endurance exercise activates the AKT and mTOR signaling pathways, which are critical for muscle hypertrophy, particularly during the recovery phase following exercise [77]. Additionally, endurance exercise upregulates vascular endothelial growth factor expression, stimulating capillary formation within skeletal muscle. This improved capillarization facilitates oxygen delivery, supports myofiber growth, and meets the metabolic demands of the muscle [78]. These mechanisms collectively explain the significant increase in myofiber CSA observed with endurance exercise. Consequently, we propose that endurance exercise may counteract CSA reduction induced by frailty syndrome, providing an effective strategy for preserving skeletal muscle health and mitigating the deleterious effects of frailty syndrome in aging populations.

### Myofiber number in frailty syndrome

Our findings demonstrate a significant reduction in myofiber number in the FS group compared to the OM group. This decline is likely driven by the combined effects of aging and high body weight, which activate apoptotic pathways in skeletal muscle [79, 80]. Specifically, aging and high body weight are associated with elevated levels of pro‐apoptotic factors such as p53 and Bax and reduced levels of anti‐apoptotic factors such as Bcl‐2, ultimately contributing to myofiber loss [80, 81, 82, 83]. Consequently, we propose that a reduction in myofiber number may serve as a histological hallmark of skeletal muscle in frailty syndrome.

After 16 weeks of endurance exercise, we observed a significant increase in myofiber number, consistent with findings from previous studies [84, 85, 86, 87]. This increase can be attributed to several molecular mechanisms facilitated by endurance exercise. First, as described above, endurance exercise enhances skeletal muscle protein synthesis, supporting the proliferation of myofibers. Additionally, calmodulin‐dependent kinases play a critical role by decoding frequency‐dependent signals, thereby promoting adaptive responses that increase myofiber number [88]. Furthermore, endurance exercise stimulates autophagy, a critical process for skeletal muscle adaptation, which facilitates the removal of damaged organelles and proteins [89]. This enhanced autophagy function not only maintains cellular homeostasis but also creates an optimal environment by freeing up space and providing essential resources to support the proliferation and regeneration of myofibers.

### MHC isoforms in frailty syndrome

MHC isoforms serve as critical markers reflecting the physiological and functional properties of skeletal muscle. Our study revealed that aging leads to a decline in the proportion of fast MHC isoforms (e.g., IIb and IIx), a finding consistent with previous studies [90, 91, 92]. However, research focusing on MHC isoforms in frailty syndrome remains scarce. Notably, our data did not demonstrate significant differences in MHC isoform distributions between the OM and FS groups. Aging is known to downregulate the expression of fast MHC isoforms and their associated genes [90, 91, 93]. Conversely, some evidence suggests that high body weight can increase the expression of fast MHC isoforms and related genes in both humans and animal models [27, 94, 95]. We hypothesize that the lack of significant changes in MHC isoform distribution in frailty syndrome may result from the opposing effects of aging and high body weight, which may neutralize each other's influence.

Our findings indicated a significant increase in the proportion of slow MHC isoforms within the PL muscle after 16 weeks of endurance exercise. This finding aligns with the inverse relationship between fiber size and oxidative capacity, as smaller myofibers are characterized by greater oxidative enzyme activity and mitochondrial density, irrespective of fiber type [96, 97]. In our data, the mean CSA of the PL muscle was smaller compared to the QD and TA muscles, which may account for the differential shifts in MHC isoform composition observed after endurance training. This could be due to enhanced oxidase activity in the PL relative to the QD and TA muscles after endurance exercise, resulting in a higher proportion of oxidative fibers and an increased percentage of slow MHC isoforms. These findings underscore the adaptability of skeletal muscle to endurance exercise, particularly in muscles like the PL, which are more predisposed to oxidative functional demands.

Overall, endurance exercise mitigates frailty‐related muscle deterioration by increasing myofiber CSA, strength, and mitochondrial function while reducing fibrosis and ECM deposition, ultimately improving mobility and reducing disability risk. These adaptations may be mediated by conserved molecular pathways, including AMPK activation, PGC‐1α signaling, and mitochondrial biogenesis, underscoring the translational relevance of exercise‐based interventions for frailty. To bridge the gap between preclinical and clinical research, future studies should integrate longitudinal clinical trials, muscle biopsy analyses, and advanced imaging techniques to validate these effects in human populations.

### Limitations

In this study, high‐resolution analysis of skeletal muscle using transmission electron microscopy (TEM) was not performed. TEM enables detailed examination of ultrastructural changes in muscle fibers and organelles, which could provide further insights into the effects of frailty syndrome in mice. Furthermore, employing Oil Red O staining would allow for a more detailed and quantitative assessment of adipose tissue infiltration within skeletal muscle, providing critical insights into the extent of lipid accumulation and its pathological effects in frailty syndrome. While we investigated the histological characteristics associated with frailty syndrome, future research should focus on assessing specific protein and gene expression patterns to deepen our understanding of the underlying molecular mechanisms in frailty syndrome.

## Conflict of interest

No conflicts of interest, financial or otherwise, have been declared by the author(s).

## Peer review

The peer review history for this article is available at https://www.webofscience.com/api/gateway/wos/peer‐review/10.1002/2211‐5463.70049.

## Author contributions

FJ: Conceptualization, data curation, formal analysis, investigation, methodology, writing – original draft. HSL: Data curation, investigation. HL: Formal analysis, methodology. J‐HK: Conceptualization, supervision, funding acquisition, writing—review and editing.

## Supporting information


**Table S1.** Selection process and positive indicators for frailty syndrome mice model.
**Data S1**. Silver‐stained bands correspond to myosin heavy chains 955 confirmation.

**Video S1.** Mouse during voluntary wheel test.

**Video S2.** Running distance monitoring using the voluntary wheel apparatus.

## Data Availability

The data used to support the findings of this study are presented here. Any further data requirements are available from the corresponding author upon request.
