# Supplementary material for: The impact of frailty syndrome on skeletal muscle histology: preventive effects of exercise
Source: FEBS Open Bio. 2025 May 5;15(8):1267–84. doi: 10.1002/2211-5463.70049 (PMC12319717; doi:10.1002/2211-5463.70049)
Supplement: Supplementary file 1 — Table S1. Selection process and positive indicators for frailty syndrome mice model. Data S1. Silver‐stained bands correspond to myosin heavy chains 955 confirmation. [file FEB4-15-1267-s002.zip › S2_(2) (1).docx]

Supplementary information 2 (S2): silver-stained bands correspond to myosin heavy chains confirmation.

To confirm that the silver-stained bands correspond to myosin heavy chains, we used an anti-MHC antibody (Myosin 4 Monoclonal Antibody, Thermo, #14-6503-82) for verification.

First, we confirmed that the myosin heavy chain's position in WB (Western Blot) matched its position in silver staining prior to separation (Figure 1).

Next, we verified that, after electrophoresis, the position of the separated myosin heavy chains remained consistent between WB and silver staining (Figure 2).

These findings conclusively demonstrate that the bands observed in silver staining correspond to the separated myosin heavy chains.


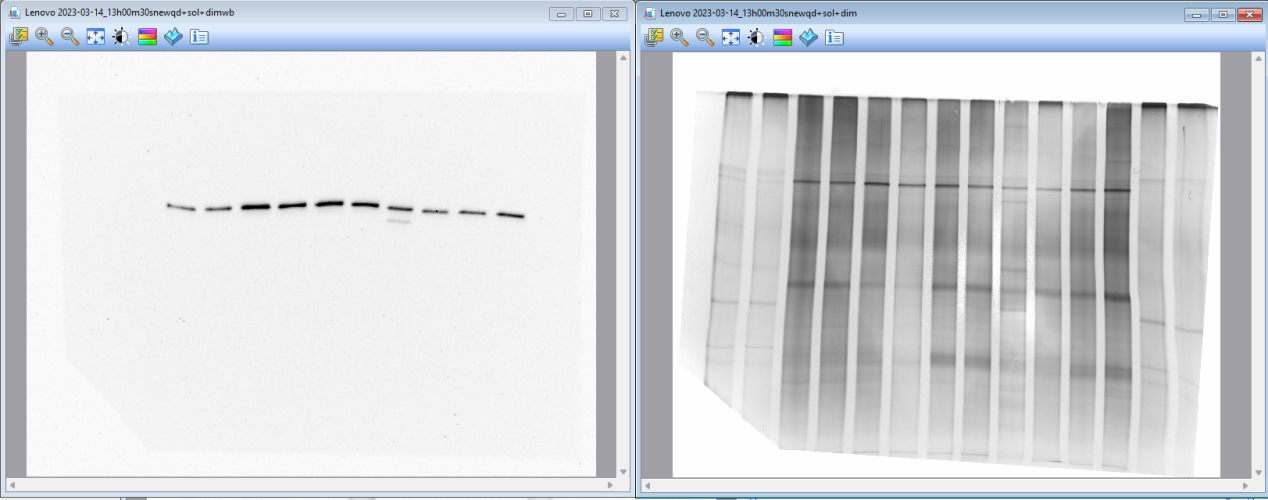


Figure 1 WB result (left) and silver staining result (right)


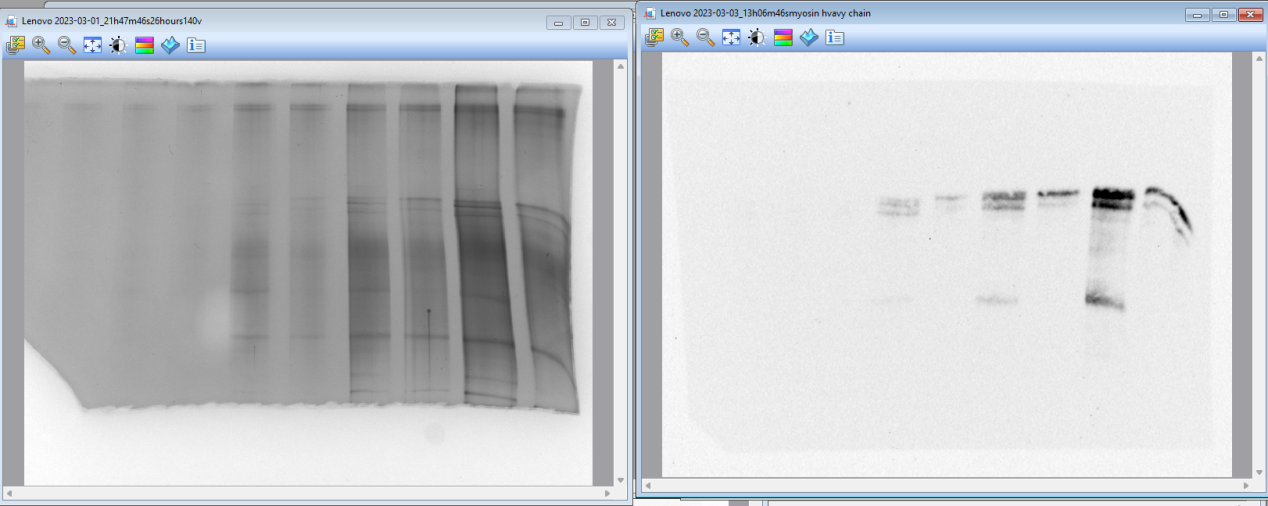


Figure 2 WB result (left) and silver staining result (right)
